# A Founder Large Deletion Mutation in Xeroderma Pigmentosum-Variant Form in Tunisia: Implication for Molecular Diagnosis and Therapy

**DOI:** 10.1155/2014/256245

**Published:** 2014-05-04

**Authors:** Mariem Ben Rekaya, Nadia Laroussi, Olfa Messaoud, Mariem Jones, Manel Jerbi, Chokri Naouali, Yosra Bouyacoub, Mariem Chargui, Rym Kefi, Becima Fazaa, Mohamed Samir Boubaker, Hamouda Boussen, Mourad Mokni, Sonia Abdelhak, Mohamed Zghal, Aida Khaled, Houda Yacoub-Youssef

**Affiliations:** ^1^Laboratoire de Génomique Biomédicale et Oncogénétique (LR 11 IPT 05), Institut Pasteur de Tunis, Université de Tunis El Manar, El Manar I, BP 74, 13 Place Pasteur 1002 Tunis Belvédère, 2092 Tunis, Tunisia; ^2^Département de Dermatologie, Hôpital Charles Nicolle de Tunis, 1006 Tunis, Tunisia; ^3^Département d'Anatomie-Pathologique Humaine et Expérimentale, Institut Pasteur de Tunis, 1002 Tunis, Tunisia; ^4^Département d'Oncologie Médicale, Hôpital Abderrahman Mami, 2080 Ariana, Tunisia; ^5^Département d'Oncologie Médicale, Hôpital La Rabta de Tunis, 1007 Tunis, Tunisia

## Abstract

Xeroderma pigmentosum Variant (XP-V) form is characterized by a late onset of skin symptoms. Our aim is the clinical and genetic investigations of XP-V Tunisian patients in order to develop a simple tool for early diagnosis. We investigated 16 suspected XP patients belonging to ten consanguineous families. Analysis of the* POLH* gene was performed by linkage analysis, long range PCR, and sequencing. Genetic analysis showed linkage to the* POLH* gene with a founder haplotype in all affected patients. Long range PCR of exon 9 to exon 11 showed a 3926 bp deletion compared to control individuals. Sequence analysis demonstrates that this deletion has occurred between two Alu-Sq2 repetitive sequences in the same orientation, respectively, in introns 9 and 10. We suggest that this mutation* POLH *NG_009252.1: g.36847_40771del3925 is caused by an equal crossover event that occurred between two homologous chromosomes at meiosis. These results allowed us to develop a simple test based on a simple PCR in order to screen suspected XP-V patients. In Tunisia, the prevalence of XP-V group seems to be underestimated and clinical diagnosis is usually later. Cascade screening of this founder mutation by PCR in regions with high frequency of XP provides a rapid and cost-effective tool for early diagnosis of XP-V in Tunisia and North Africa.

## 1. Introduction

Xeroderma pigmentosum (XP) is an autosomal recessive cancer prone disease characterized by sensitivity to ultraviolet rays (UVR). XP patients are consequently predisposed to develop skin and eyes cancers [1]. This disease is caused by inherited mutations in DNA repair genes encoding proteins that protect cells from UV-induced damage. XP is genetically heterogeneous with seven XP complementation groups (XP-A to XP-G) defective in nucleotide excision repair (NER) pathway and an additional “variant” form (XP-V) with normal NER but a deficient translesional synthesis.

XP-V patients have a relatively milder phenotype with a late onset of symptoms and delayed progression. Typically, XP-V patients do not have ocular or neurological abnormalities [2]. Many studies suggest that this form of XP is underdiagnosed [3, 4]. Therefore, XP-V patients represent only 20 to 30% of all XP cases [5].

Cells from XP-V patients are extremely hypermutable after exposure to UV due to the deficiency of pol eta [6, 7]. The DNA polymerase eta (*η*) normally catalyzes translesion synthesis (TLS) by incorporating dAMP opposite thymine residues of a cyclobutane thymine dimer (CPD) [8, 9]. In the absence of pol eta, the highly error-prone pol iota undertakes this bypass function resulting in the accumulation of UV-induced mutations and an increase in the susceptibility to skin cancer [10, 11].

Pol eta is encoded by the* POLH* gene, the human homolog of yeast Rad30 [9]. Pol *η* plays an important role in preventing genome instability after UV or cisplatin-induced DNA damage [12]. Chemoresistance of cancer to cisplatin treatment is due in part to human Pol *η*. Crystal structures of hPol *η* complexed with intrastrand cisplatin identified a hydrophobic pocket as a potential drug target for reducing chemoresistance [13].

More than 60 mutations have been identified in the* POLH* gene in cell lines derived from XP-V patients from different geographic locations, mainly Russia-Armenia, Scotland, Lebanon, Iran, Belgium, France, Japan, USA, Europe, Asia, Cayman Islands, Turkey, Israel, Germany, Korea, Algeria, and Tunisia [2–4, 9, 14–23].

In our study, we surveyed* POLH* mutations in 16 Tunisian patients with late onset of XP in order to assess the causative mutations of this disorder and to develop a rapid molecular diagnostic test.

## 2. Patients and Methods

### 2.1. Patients

Sixteen suspected XP-V patients belonging to ten consanguineous Tunisian families originated from different regions of Tunisia were investigated (Table 2). Their age was ranging from 4 to 50 years.

### 2.2. Methods

Written informed consent was obtained from all available family members or from parents of minor children. Families were interviewed using a structured questionnaire to collect information about family history, consanguinity, affected members, and associated diseases. DNA was isolated from peripheral blood leukocyte using salting-out method [24] or Qiagen kit DNA extraction.

#### 2.2.1. Genetic Analysis

To confirm linkage to* POLH* gene, available family members were genotyped using two polymorphic microsatellite markers spanning a 0.4 Mb interval near to* POLH* locus (cen-D6S207 and D6S1582 (*POLH*-)tel) as previously described [4]. Microsatellite markers were selected from the genetic maps available on NCBI browsers and the CEPH genotype database (http://www.cephb.fr/en/cephdb/) on the basis of their heterozygosity percentage and closeness to the* POLH* gene. Genotyping was performed as described elsewhere [25].

#### 2.2.2. PCR Long-Range

On absence of amplification of* POLH* exon 10, long PCR was performed using the Expand Long Template PCR System Kit (Expand Long Range dNTPack 700 units/*μ*L Roche). PCR was performed using different primers (Table 3). The PCR program included 92°C for 2 min, 10 cycles of 92°C for 10 sec, 60°C for 15 sec, 68°C for 10 min, and 20 cycles of the same program except that the extension step was extended by 20 sec per cycle. PCR products were run on 1% agarose gel with the DNA ladder 1 kb molecular size marker (GeneRulerTM).

#### 2.2.3. Bioinformatic Analysis

As several genomic rearrangements are commonly caused by recombination events induced by repetitive elements present in the human genome, the genomic sequence of the* POLH* gene (NG_009252.1) was obtained and analyzed from chr6: 43578281 to 43581387 position corresponding to exon 9 to exon 11 region. Screening for repetitive elements was performed using the RepeatMasker software available at http://www.repeatmasker.org/ (Table 1).

#### 2.2.4. Sequencing and Mutation Analysis

Long range PCR products were directly sequenced using the ABI 3130 Genetic Analyzer by two pairs of primers (Table 3). Mutation analysis and breakpoints of the deletion were determined as the last nucleotide showing sequence identity between wild and mutated sequences (Figure 3).

#### 2.2.5. Mutation Nomenclature

The genomic reference of the* POLH *gene NG_011763.1 was used to annotate the deletion according to the HGVS version 2.0 (Mutalyzer 2.0.beta-26).

## 3. Results

### 3.1. Clinical Findings

In this study, we investigated sixteen patients with late onset of XP features. These patients belong to ten consanguineous families from different Tunisian geographic areas (Table 2). For all patients, skin hyperphotosensitivity to UVR began at a mean age of 4 years. The mean age at onset of the first skin cancer was 24 years. Nonmelanoma skin cancer (NMSC) occurred in only 10 patients. At least, 3 among them developed only basal cell carcinoma (BCC) and 5 developed squamous cell carcinoma (SCC) combined with BCC (Table 2).

### 3.2. Genetic Analysis

**Figure 1:**
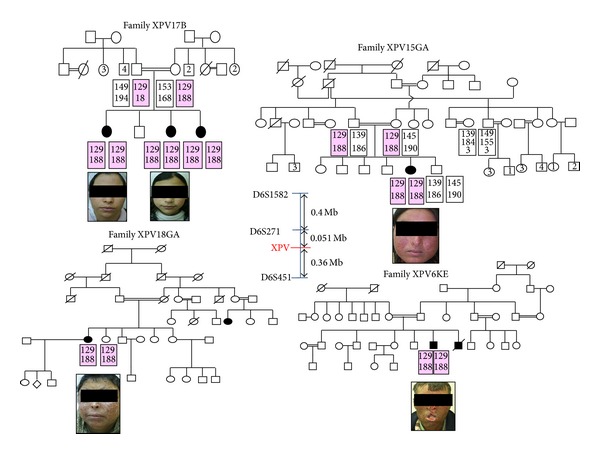
Pedigree and haplotype analysis for the XPV families (the disease haplotype is indicated by shading) and clinical photograph of each affected patient.

The genetic examination of XP-V patients was initially assessed through routine procedures, which involved genotyping of all consanguineous XP-V patients and available related individuals. Haplotype analysis showed homozygosity for the closest two markers to* POLH* gene, D6S1582 and D6S271, with a founder haplotype (129–188) in all investigated patients (Figure 1).

### 3.3. PCR Long-Range

**Figure 2:**
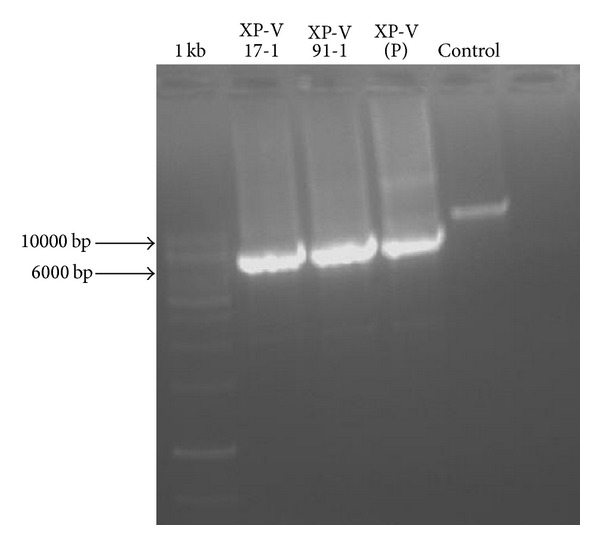
Agar gel electrophoretic analysis of the PCR* POLH* gDNA of exon 10 and its intronic boundaries showed difference in the size between affected individuals (XPV17B-1 and XPV91) compared to healthy parents (XPV(P)) and a healthy control. (Marker: 1 kb DNA ladder molecular size marker (GeneRuler).)

DNA samples from these patients repeatedly failed to yield PCR amplification products for exon 10. Therefore, we assumed the presence of a genomic deletion spanning exon 10 (del exon 10). As del exon 10 was previously described in Italian patient at the genomic DNA level with 2.7 Kb deletion [2], we first screen for this deletion. Therefore, screening of this deletion by PCR did not yield any amplification product confirming that there are different breakpoints involved in our XP-V patients. In order to identify the deletion size, we amplified the sequence between exon 9 and exon 11 using primers POLH10ΔF and POLH10ΔR showed in Table 3. Long range PCR revealed *a* ≈ 6 kb product for XP-V patients versus *≈*10 kb in control individual corresponding to approximately 4 kb size deletion (Figure 2).

### 3.4. Bioinformatic Analysis

Screening of repetitive elements present in exon 9 to exon 11 using repeat masker software revealed that 51.44% (4814 pb) of the sequence was interspersed repeat sequences. Among them, 11 SINE Alu sequences spanned a region of 2908 bp (31.08% of all the sequence) and 3 LINE sequences spanned a region of 1789 bp (19.12% of all the sequence). These Alu sequences are predicted to promote the occurrence of large deletions (Table 1).

### 3.5. Mutation Screening

**Figure 3:**
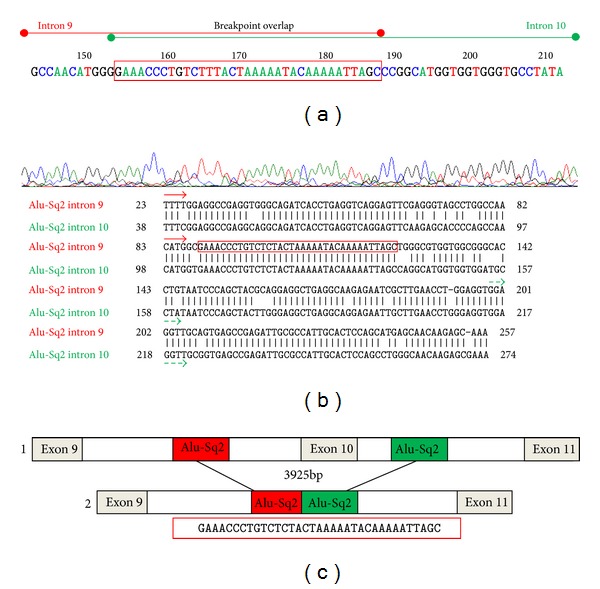
Characterization of the deletion breakpoints. (a) Electropherogram demonstrating the junction fragment resulting from the large deletion in the XP-V patients. Partial representation of introns 9 and 10 with the 35 bp breakpoint overlap framed in red. (b) Nucleotide sequence alignment of the genomic sequence of introns 9 and 10 of the* POLH* gene. Short vertical lines indicate matched bases between both introns. (c) Schematic representation of the deletion breakpoints and their flanking Alu Sq2 elements. (1) represents a normal gDNA fragment and (2) schematizes the mutated gDNA with a deletion of 3925 bp.

In order to detect the breakpoints with accuracy, two internal primer pairs were designed to sequence introns 9 and 10 across the deletion (Table 3). Direct sequencing and analysis of the 6 kb PCR product (Figure 2) of XPV17 and XPV91 patients using primers POLHdelF and POLHdelR revealed that both the 5′ and 3′ breakpoints were located within homologous Alu Sq2 (class SINE (short interspersed elements, family Alu)) elements in introns 9 and 10 of* POLH* gene (Figure 3). This deletion* POLH *NG_009252.1: g.32438_36363del3926led to the loss of exon 10 (c.1370-2567_1539+1188del3925). This mutation has likely resulted from Alu-Alu equal homologous recombination.

### 3.6. Screening of Deletion by PCR

After identification of the deletion breakpoints in two patients (XPV91 and XPV17), we screened the following patients for this deletion by PCR using primers POLHdelF and POLHdelR showed in Table 3. In all patients, we found a product of 500 pb versus 4500 pb in virtual PCR. We then confirmed the presence of the same breakpoints by direct sequencing.

For individuals at a heterozygous state, we confirmed their profiles by two PCRs. The presence of one allele of exon 10 was confirmed using XPV10F and XPV10R primers and the absence of exon 10 on the other allele was confirmed using POLHdelF and POLHdelR primers.

## 4. Discussion

We report 16 cases with NMSC, BCC, and SCC that occurred with a mean delay of 24 years after XP diagnosis. Five of our patients (XPV6KE, XP18G XPV20G, XPV43-1, and XPV53Z) had been treated by skin radiotherapy (Table 2). After cancer treatment, many NMSC appeared. For example, XPV6KE died after frontal tumor metastasis and XPV18G experienced a metastasis after recurrence on the right cheek. These consequences may be explained by the significant role of pol eta in cancer radiotherapy response. Pol eta-deficient cells are resistant to ionizing radiation. This radioresistance results from the increased reparation of double strand breaks by homologous recombination repair system (HR) [26]. While for chemotherapy, previous studies demonstrate that pol eta-deficient cells are very sensitive to cisplatin and oxaliplatin and particularly for agents which exert their activities by blocking DNA replication forks [27]. Among the roles of pol eta is repairing lesions induced by cisplatin. Consequently, systemic chemotherapy using cisplatin will attack healthy cells and induce novel cancers on absence of pol eta. This type of chemotherapy may be very dangerous for XP-V patients. Knowing this important role of pol eta, mutation screening of* POLH* gene in patients with SCC or BCC could have an impact in guiding treatment choice.

Previous studies showed two specific mutations (c.1568_1571delGTCA and c.660+1G>A) in three XP-V Tunisian patients [4, 20]. Deletion of exon 10 has been previously described at the cDNA level in XP-V patients from different geographic origins. It was found at homozygous state in two Algerian (XP62VI and XP75VI) and in one American (XP139DC) and at heterozygous state in one Tunisian (XP28VI) XP-V patients [3, 16]. Also,* POLH* del exon 10 has been described at genomic level in one Italian patient with 2.7 Kb deletion occurring between two poly (T) sequences [2] and in one Algerian XP-V patient with 3.763 bp deletion [22]. We report here a novel breakpoint of del exon 10,* POLH *NG_009252.1: g.32438_36363del3926, that presents in 16 XP-V Tunisian patients belonging to 10 unrelated families. This deletion can be screened by a simple PCR without confirming by sequencing. This rapid tool may facilitate molecular investigation of XP-V patient.

This mutation is probably a founder variation because it was carried by a particular haplotype (129–188 or 129–186). Del exon 10 is common in the world and probably it may be due to different founder effects. Repetitive sequences are the primary candidates to generate stable abnormal secondary structures producing large deletion during replication [28]. Alu elements are normally located within introns and 3′ untranslated regions of genes, which are considered mutational “hotspots” for large gene rearrangements [29]. Large deletions in* POLH* gene have been previously described in exons 5 and 6 [3, 16]. Similar founder mutations in the* POLH* gene have been reported in other populations such as Japanese and Korean. Therefore, 87% of the Japanese XP-V patients shared one of the four founder mutations described in Japan [3, 18].

## 5. Conclusion

The presence of this founder mutation, reported in our study, could simplify genetic screening of XP patients in Tunisian population by implementing presymptomatic tests and hence early UV protection. Before treatment of patients' skin cancers, XP status should be verified to avoid cancer recurrence. It is also important to consider the possible existence of such large deletion at heterozygous state. Consequently, we propose systematic screening of this mutation in all XP-V patients by two PC reactions; the 1st will amplify exon 10, while the 2nd will amplify across deletion breakpoints. After confirmation at a large scale in XP Tunisian patients, the test will be proposed for patients from Southern Mediterranean and Middle East countries.

## Figures and Tables

**Table 1:** Sequence analysis of repetitive elements of the 9358 bp sequence of *POLH* gene: 43572521–43581878 using repeat masker Software.

score	% div.	% del.	% ins.	Query sequence	Position in query-			C +	Matching repeat	Repeat class/family	-Position in repeat (left) end begin			linkage
					begin	end	(left)	+	repeat	class/family	begin	end	(left)	id/graphic
1692	13.5	0.4	0.4	chr6:POLH:43572521+43581878	657	893	(8465)	C	AluJr	SINE/Alu	(2)	310	74	1
2266	10.4	0.3	4.3	chr6:POLH:43572521+43581878	925	1240	(8118)	C	AluSq	SINE/Alu	(9)	304	1	2
658	26.3	8.6	4.6	chr6:POLH:43572521+43581878	1267	1581	(7777)	C	L1MA8	LINE/L1	(25)	6266	5940	3
2432	12.0	0.0	0.0	chr6:POLH:43572521+43581878	1644	1944	(7414)	C	AluSx1	SINE/Alu	(10)	302	2	4
3529	14.9	6.9	4.2	chr6:POLH:43572521+43581878	1958	2302	(7056)	C	L1MB8	LINE/L1	(0)	6178	5821	5
2464	10.8	0.0	0.0	chr6:POLH:43572521+43581878	2303	2609	(6749)	C	AluSc5	SINE/Alu	(2)	307	1	6
3529	16.1	7.0	4.4	chr6:POLH:43572521+43581878	2610	3080	(6278	C	L1MB8	LINE/L1	(342)	5820	5323	5
2287	9.2	5.5	0.0	chr6:POLH:43572521+43581878	3081	3372	(5986)	+	AluSq2	SINE/Alu	1	308	(5)	7
1739	17.8	10.7	1.8	chr6:POLH:43572521+43581878	3373	3510	(5848)	C	L1MB8	LINE/L1	(829)	5333	5170	5
810	18.7	1.2	7.0	chr6:POLH:43572521+43581878	3744	3909	(5449)	C	AluJo	SINE/Alu	(19)	293	137	8
535	29.6	9.7	1.4	chr6:POLH:43572521+43581878	3990	4509	(4849)	+	L2a	LINE/L2	(2804)	3365	(61)	9
2528	10.0	0.0	1.0	chr6:POLH:43572521+43581878	5271	5581	(3777)	C	AluSx1	SINE/Alu	(4)	308	1	10
13	10.0	5.9	2.8	chr6:POLH:43572521+43581878	5582	5615	(3743)	+	(TCTTTA)n	Simple_repeat	1	36	(0)	11
684	5.6	0.0	0.0	chr6:POLH:43572521+43581878	6162	6233	(3125)	+	AluSq10	SINE/Alu	1	72	(241)	12
2510	10.3	0.0	0.0	chr6:POLH:43572521+43581878	6991	7300	(2058)	+	AluSq2	SINE/Alu	1	310	(3)	13
2679	7.0	0.0	0.0	chr6:POLH:43572521+43581878	7564	7863	(1495)	C	AluSq	SINE/Alu	(13)	300	1	14
2503	8.4	0.7	0.0	chr6:POLH:43572521+43581878	8018	8313	(1045)	C	AluSg	SINE/Alu	(11)	299	2	15
214	24.7	18.6	1.0	chr6:POLH:43572521+43581878	8598	8683	(675)	+	MER5A	DNA/hAT-Charlie	2	102	(87)	16
196	16.1	0.0	0.0	chr6:POLH:43572521+43581878	8684	8714	(644)	+	MER5A	DNA/hAT-Charlie	(159)	189	(0)	17

**Table 2:** Clinical features of Tunisian XP-V patients.

Patients	Affected patients	Sex	Age (years)	Age at onset of the 1st XP macules erythema (years)	Geographic Origin	Age at onset of 1st Tumor in years (Number of tumors)			Photophobia	Radiotherapy	Tumor post Radiotherapy
						BCC	SCC	Other tumors			
XPV6KE	1	M	47 (died at 50)	4	Kef	22 (6)	20 (12)	—	+/−	+++	+++
XPV15GA	1	F	18	4	Gafsa	15 (2)	16 (2)		+/−		
XPV17-1 B	3	F	17	4	Bizerte	0	0	0	—		
XPV17-2 B		F	11	4		0	0	0	—		
XPV17-3 B		F	4	5		0	0	0	—		
XP18G	1	F	43	4	Gafsa	16 (8)	21 (3)	KA	+/−	+++	+++
XPV20G	3	F	31	5	Gafsa	ND (>10)	ND (>2)			+++	+
XPV43-1	7	M	ND	5	Zaghouan	38 (8)	23 (4)	KA and Actinic Keratosis	+/−	+ (local)	—
XPV43-2		F	45?	5		41 (1)			+/−		
XPV48G	1	F	46	6	Gafsa	0	47 (1)		+/−		
XPV53Z	1	M	50	7	Fahs Zaghouan	37 (4)				+ (local)	+/−
XPV79-1	3 (1died)	F	13	3	Tozeur/Gafsa	10 (3)	—	KA++	+/−		
XPV79-2		F	18	3				KA	—		
XPV91-1	3	F	29	6	Tozeur			Actinic Keratosis	—		
XPV91-2		F	21	6					—		
XPV91-3		F	24	6					—		

SCC: spino cell carcinoma; BCC: basal cell Carcinoma; KA: kerathoacantum; (+/−) moderate phenotype; (—) absence; (+++) several.

**Table 3:** Complete list of primers used to gDNA amplification of exon 10 and its intronic boundaries.

Name	Sequence 5′→3′	Annealing Temperature (°C)	Suspected PCR products size (bp)	PCR Product size for XP-V patients (bp)
XPV10F	CCTGGTTCTTTTAATTTCCTCTCCTG	55	459	—
XPV10R	CATTTACCCTTTACCTCATTGAAGGAC			

XPV del 10 F	TCATTTGTGCTGTCCTGTTC	60	3012	—
XPV del 10 R	GGTTGCAGTGAGCGGAGATT			

Del ex10 LR-F	AGGTCCTCCCTAGTTACCCTATCACAGCAG	60	4105	—
Del ex10 LR-R	ACTACCTAACCCTGACTGACTTACCACTCTGG			

POLH10ΔF	AGTGGGTAGGTTTTGGTAGCTGTGGAAG	60	9358	*≈*6000 bp
POLH10ΔR	GGACACACCCTGGATACTCTGTTGGTAA			

POLHFΔ	ACCTTGGAGTATAATTTCTGGGTCA	59	5212	*≈*1000 bp
POLHRΔ	GTCATAAAGTTCCTCATTGTGTCTAA			

POLHdelF	CATGTGCTTGTTGGACATTTG	60	4526	*≈*500 bp
POLHdelR	GGTTTCATGCTTTGGGACAG			
